# Topical clobetasol propionate 0.025% oral gel in the treatment of oral lichen planus: a double-blind, randomized placebo-controlled trial

**DOI:** 10.1186/s12903-026-08599-7

**Published:** 2026-05-20

**Authors:** Fredrik Gränse, Liv Kroona, Anna Truedsson, Cecilia Larsson Wexell, Bengt Götrick

**Affiliations:** 1 Department of Oral and Maxillofacial Surgery and Oral Medicine, Faculty of Odontology, Malmö University, Malmö, SE-205 06 Sweden; 2 Department of Oral Pathology, Faculty of Odontology, Malmö University, Malmö, Sweden; 3 Department of Oral and Maxillofacial Surgery, Skåne University Hospital, Lund, Sweden; 4 Department of Orofacial Medicine, Faculty of Odontology, Malmö University, Malmö, Sweden

**Keywords:** Oral lichen planus, Topical clobetasol propionate, Randomized controlled trial

## Abstract

**Background:**

Oral lichen planus (OLP) is a chronic immune‑mediated inflammatory disorder affecting the oral mucosa and characterized by recurring exacerbations and remissions. Complete healing is uncommon. Currently, there is no established curative therapy for OLP. Management strategies are aimed at alleviating clinical symptoms by reducing the underlying inflammatory response. Although some effect of topical corticosteroids has been reported, the level of evidence remains low, as few studies are randomized and placebo controlled. This study evaluated the efficacy of clobetasol compared with placebo in the treatment of symptomatic OLP and sought to determine whether once-daily is as effective as twice-daily administration.

**Methods:**

Patients with symptomatic OLP were randomly allocated to three treatment groups: clobetasol propionate 0.025% oral gel twice daily (Active 2, *n* = 22), clobetasol propionate 0.025% oral gel once daily and placebo oral gel once daily (Active 1, *n* = 26) and placebo oral gel twice daily (Placebo, *n* = 25). All three groups were instructed to rinse with the assigned oral gel for 1 min twice daily for 4 weeks. The primary outcome was the Oral Disease Severity Score (ODSS). Secondary outcomes were the visual analogue scale (VAS) score for pain, the VAS score for burning sensation, the Oral Health Impact Profile (OHIP-14), and the Global Rating of Change (GRC).

**Results:**

In both intervention groups (Active 2 and 1), improvement in the ODSS (*p* < 0.05) and the GRC (*p* < 0.05) after 4 weeks was significant compared with control group (Placebo). No significant differences were observed between the intervention groups in ODSS and GRC. Nor were any significant differences in the pain score, the burning sensation score, or the OHIP-14 noted between the intervention groups and the control group. Adverse events were mild with no significant differences between the intervention groups and the control group. Despite receiving antifungal prophylaxis, 27% of the participants had oral candidiasis at the end of treatment.

**Conclusions:**

The results suggest that topical clobetasol propionate 0.025% oral gel is more effective than placebo in improving disease severity (ODSS) and global change (GRC) in patients with symptomatic OLP. The results also suggest that once-daily treatment is as effective as twice-daily treatment.

**Trial registration:**

This trial was registered at Clinical Trials.gov on April 20, 2020, under registration number NCT04364555.

## Introduction

With a global prevalence of approximately 1%, oral lichen planus (OLP) is a frequent condition in oral medicine practice [1]. The clinical and histological signs of OLP include a chronic autoimmune-like inflammation in the oral mucosa driven by a T-cell mediated immune response. The underlying reason for this reaction is still uncertain, but may involve presentation of altered self-antigen by basal keratinocytes [2]. OLP usually debuts at an age of 40–60 years and is more common in women [3]. Around two-thirds of patients diagnosed with OLP experience symptoms [4]. The severity of these varies, from mild burning sensations to long periods of pain and severely affected quality of life [5]. OLP is characterized by recurring exacerbations and remissions of symptoms. Complete resolution of the condition is uncommon.

Although OLP can affect any region of the oral mucosa, it is most frequently observed on the buccal mucosa, tongue and gingiva. Lesions are typically white or a combination of white and erythematous areas. White components appear as papular, reticular or plaque-like patterns, and erythematous components as erosive, ulcerous or bullous changes [6]. The characteristic clinical appearance, supported by the patient’s medical history, is usually sufficient for diagnosis; a biopsy may confirm histopathological features and rule out dysplasia or malignancy. Asymmetrical or solitary lesions, and those induced by drugs, contact allergens, or systemic conditions are classified as oral lichenoid lesions [7]. The risk of malignant transformation of OLP is low, approximately 0.28% per year, but long-standing untreated ulcerous lesions are considered a potential risk factor [8].

Although many interventions have been tested, none have so far been shown to permanently cure OLP [9]. Current treatments aim to alleviate the patient’s symptoms by reducing the inflammatory process [10]. The first step is to improve oral hygiene, a measure that can provide symptomatic relief for some patients [11]. Patients whose symptoms persist despite this intervention may be considered for medical treatment.

A Cochrane review from 2020 [12] evaluated the treatment of symptomatic OLP with local corticosteroids. The review found low-certainty evidence that local corticosteroids reduce pain more effectively than placebo, and the three randomized, placebo-controlled studies included showed inconclusive results regarding clinical improvement and adverse effects. In these studies, fluocinolone 0.025% [13], triamcinolone 0.1% [14], and clobetasol 0.05% [15] were investigated. Corticosteroids are nevertheless considered the first-line treatment for symptomatic OLP.

Among the corticosteroids commonly used to treat OLP, clobetasol (clobetasol propionate) is the most potent (Class 1, super potent). Not surprisingly, studies report better efficacy but higher rates of adverse reactions compared with other corticosteroids [16]. Examples of adverse effects include oral candidiasis [17, 18] and glucocorticoid-induced adrenal insufficiency [19]. The most studied treatment with clobetasol is 0.05% ointment 2–4 times daily [20]. Further studies are needed to assess the efficacy of clobetasol in the treatment of OLP and to determine the lowest effective dosage as well as the optimal treatment regimen. The aim of this study was to evaluate the effects of 0.025% clobetasol oral gel rinses on disease severity and self‑reported symptoms in patients with symptomatic OLP, and to determine whether once‑daily administration is as effective as twice‑daily dosing.

## Materials and methods

### Study design

This study was performed as a three-arm prospective, randomized, placebo-controlled, double-blind clinical trial involving patients with symptomatic OLP. The Medical Products Agency in Sweden (Daybook no. [Dnr]: 5.1-2020-133) and the Regional Ethics Review Board in Lund, Sweden (Dnr: 2019–06541; Dnr: 2024-03429-02) approved the study, which was conducted in accordance with the Declaration of Helsinki [21]. The study protocol was registered in the European Union Drug Regulating Authorities Clinical Trials (EudraCT) database (EU no.: 2018-004222-28) and at Clinical Trials.gov (NCT04364555) on April 20, 2020. The study was conducted and reported in accordance with the CONSORT 2025 guideline for reporting randomised trials [22].

### Patients

Consecutive patients referred to the Department of Oral and Maxillofacial Surgery and Oral Medicine at the Faculty of Odontology, Malmö University (Sweden), or to the Department of Oral and Maxillofacial Surgery at Skåne University Hospital in Lund (Sweden), were enrolled between June 2020 and July 2024. To be eligible for initial inclusion, patients were required to present with clinical signs consistent with OLP and report oral symptoms such as pain or burning sensation. Patients were included, clinically assessed, treated, and evaluated by the same dental specialist in oral medicine (FG, first author). Medical and dental histories were recorded. To histologically verify the OLP diagnosis, a 5-mm punch biopsy of a representative region with white striae and erythematous areas in one of the oral lesions was taken. Patients requiring improved oral hygiene were provided with suitable information and instructions.

To identify any presence of candida infected OLP lesions at baseline and after 4 weeks of treatment, mucosal smears of lesions were retrieved with a sterile raspatory and fixed to a glass slide with alcohol. The samples were analysed at the Department of Oral Pathology, Malmö University for presence of hyphae or pseudohyphae after PAS staining. Patient eligibility for final inclusion in the study required a diagnosis of OLP according to the modified WHO criteria of OLP [7] and patient-rated mucosal symptoms with a visual analogue scale (VAS) score (see below) of 1 or more for pain or burning sensation. In addition, the Silness-Löe index [23] was used to assess oral hygiene; patients with an index > 2 were excluded. The guidelines of Papapanou et al. [24] were used to assess periodontitis; patients with stage III or IV (e.g., clinical loss of supporting tissues > 5 mm, pocket depth > 6 mm, furcation involvement grades 2–3) were excluded. The rationale was that untreated and severe periodontal inflammation and high bacterial load may aggravate oral mucosal inflammation and affect the treatment outcome negatively [25]. Patients with previous oral malignancy were excluded as consequences of cancer treatment could affect clinical appearance of the oral mucosa as well as salivary secretion and perception [26]. Table 1 presents the inclusion and exclusion criteria. All patients received detailed information about the study procedures and signed informed-consent forms.

**Table 1:** Eligibility criteria for patients with suspected oral lichen planus (OLP)

Inclusion criteria	Exclusion criteria
• Clinical signs and symptomatic OLP	• Do not fulfil the WHO criteria for OLP
	• Ongoing antibiotic treatment
• Age ≥ 40 years	• Ongoing treatment with cortisone or any other immunomodulant medication
	• Hypersensitivity to clobetasol propionate
	• Periodontitis stage III and IV
	• Inferior and unimproved oral hygiene
	• Hypersensitivity to nystatin
	• Pregnant or lactating
	• Previous or current intraoral malignancy
	• Active participation in another drug study
	• Free of symptoms before study start
	• Declined to participate

*Abbreviations*: *WHO* World health organization

### Interventions

Patients were randomly allocated to three groups to receive a twice-daily treatment: Active 2 – clobetasol propionate 0.025% oral gel (APL Pharma Specials, Box 5071, SE-141 05, Kungens kurva, Sweden) twice daily; Active 1 – clobetasol propionate 0.025% oral gel once daily and a placebo oral gel (APL Pharma Specials) once daily; and Placebo (control group) – a placebo oral gel twice daily. All groups were instructed to rinse their mouths with 5 ml of the assigned gel for 1 min, morning and evening after oral hygiene, for 4 weeks. Patients were instructed to record their daily doses on a calendar. Compliance was further evaluated by documenting the amount of remaining gel in the medication containers. All patients received topical antifungal prophylaxis with Nystatin (Nystatin Orifarm, oral suspension 100,000 IE/ml, Orifarm Generics AB, Box 56048, SE-102 17, Stockholm, Sweden) 1 ml 4 times daily throughout treatment. After the study period, patients were followed up and managed as needed in accordance with established treatment protocols for OLP.

### Outcome measures

The following outcome measures were recorded at baseline (before the first treatment), and at 2 and 4 weeks of treatment.

#### Primary outcome

The Oral Disease Severity Score (ODSS) [27, 28] has been validated for OLP [29] and was chosen as the primary outcome measure for this study. The ODSS divides the oral mucosa into 17 sites. At each site, the extent of mucosal involvement (site score) and the severity of the lesions (severity score) were assessed. An activity score for each site was obtained by multiplying the site and severity scores. The total score was calculated by adding the total site score, the total activity score, and the patient’s perceived pain (pain score, see below), a maximum possible score of 106. All scores were recorded at each visit and documented in a standardized way with 13 photos. Prior to the study, examiner calibration was performed by grading photographs of OLP lesions according to the ODSS together with an experienced colleague.

#### Secondary outcomes

The secondary outcome measures were self-report instruments. They consisted of a pain score [30] and a burning sensation score [31], the 14-item Oral Health Impact Profile (OHIP-14) [32]; and a Global Rating of Change (GRC) assessment [33].

A VAS was used to assess pain and burning sensation. The patient recorded perceived pain and burning sensation associated with the OLP lesion by indicating the severity on a 0–10 VAS. The endpoints for both conditions were 0, which corresponded to no symptoms and 10, which corresponded to worst imaginable symptoms. The pain score was also used in calculating the ODSS.

Oral health-related quality of life was evaluated using the validated Swedish version of the OHIP-14, a 14-item self-report questionnaire [34]. Each item was rated on a scale ranging from ‘never’ (0) to ‘very often’ [4], and an overall score in the range of 0–56 was calculated.

Before assessing pain, burning sensation, and the OHIP-14 questions, patients were reminded that their responses concerned only symptoms or experiences from the preceding week. The GRC, however, was used to measure patient perceived change related to the intervention. At the 2- and 4-week follow-ups, patients were asked to score perceived changes in their OLP symptoms since baseline. Because the GRC records change due to treatment, patients were not asked to assess it at baseline. The GRC used in this study was a 7-step scale ranging from − 3 to 3, with − 3 indicating much worse, 0 indicating unchanged, and 3 indicating much better. The evaluation of the GRC compared the proportion of patients noting improvement in any form (1-3), to those recording much worse to no change (-3–0).

### Adverse effects

At both follow-up visits, patients were asked about any adverse effects related to the treatment. Any reported adverse effects were registered.

### Sample size calculation

The primary outcome measure was the ODSS. Sample size calculation was based on the previous study by Lee et al. [31] that reported a reduction in ODSS by 55% after 2 weeks of treatment, from 9.8 ± 4.6 to 4.4 ± 3.6 and an assumption of a placebo effect of 20%. With a type I error set to 0.05 and power set to 80%, 20 patients per group were needed. To compensate for potential dropouts, inclusion of 90 patients (30 for each group) was the desired aim.

### Randomization, allocation concealment and blinding

A list of randomized allocations with a block size of 6 was generated using the program Design (Trombult Programming AB, Stockholm, Sweden). The manufacturer (APL Pharma Specials) randomized and packaged the test and placebo products in identical medication containers. The containers were numbered according to the randomization list and distributed to the study sites. Randomization and allocation were carried out so that both patients and the examiner were blinded to treatment. Further, the randomization list remained sealed until all data had been collected and compiled.

### Statistics

All data analyses were performed using the IBM^®^ SPSS^®^ software package 30.0.0.0(172). The baseline variables in the two intervention groups (Active 2 and 1) and the control group (Placebo) were compared using Pearson’s Chi Square test or the Fisher-Freeman-Halton Exact Test. The one-way ANOVA was used to compare differences in age between the groups. Differences in adverse effects between groups were calculated with Pearson’s chi square test. Mean ODSS, pain, and burning sensation and means of the overall OHIP-14 scores were evaluated with the one-way ANOVA followed by Tukey’s post hoc test. Changes in the GRC between the two follow-ups were examined across groups using the Fisher-Freeman-Halton Exact Test. The significance level was set at *p* < 0.05.

## Results

**Fig. 1:**
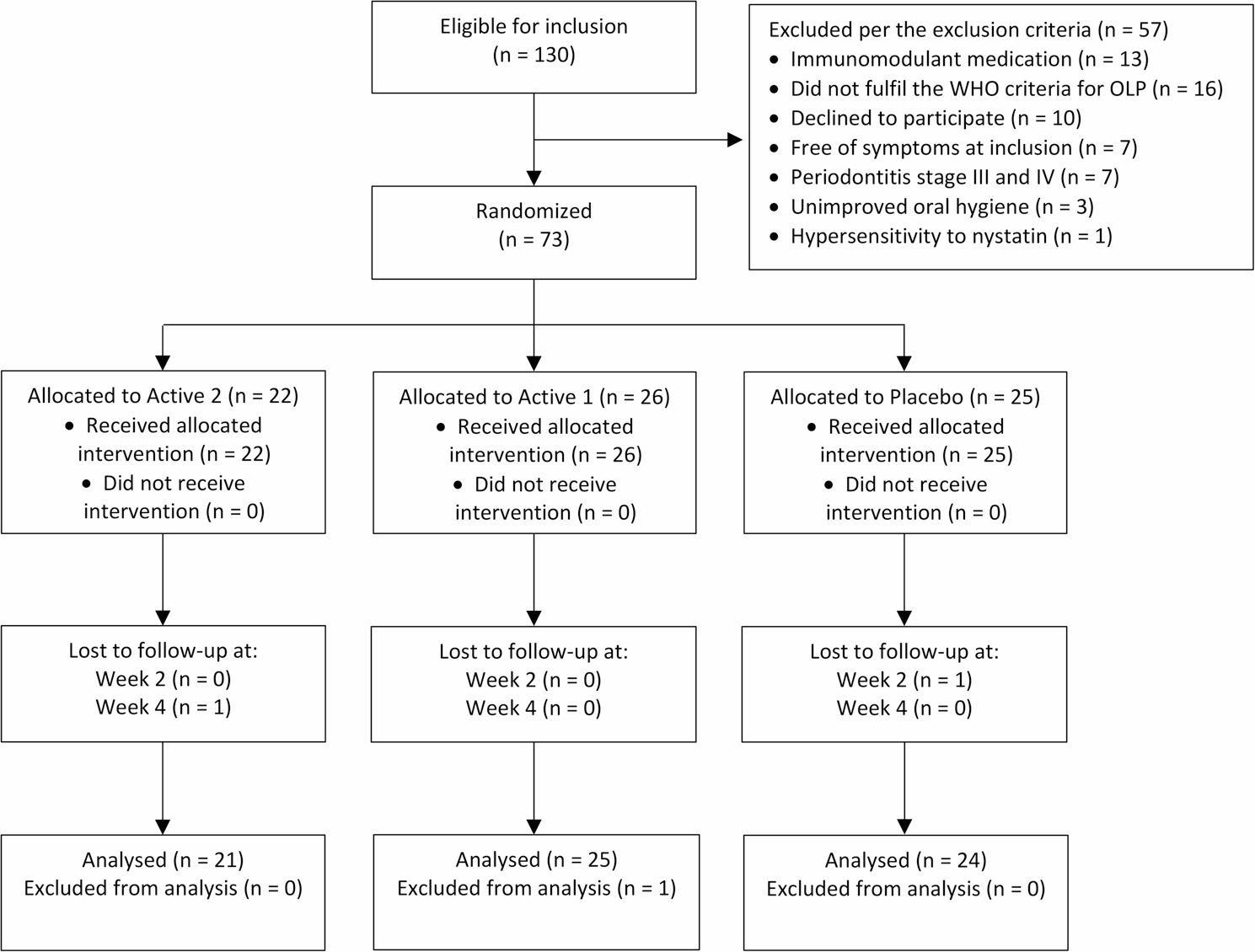
Flow diagram according to CONSORT 2025. Abbreviations: OLP, Oral lichen planus; WHO, World health organization

Of 130 patients assessed for eligibility, 73 were randomized to one of the three treatment groups. Two patients were excluded during the study because of illness unrelated to the OLP and its treatment. One patient was excluded from analysis since it later became apparent that the patient had contact allergy to mercury. With 70 included and analysed patients, the predetermined statistical power was reached, and it was decided to stop inclusion. Figure 1 illustrates patient flow. One patient in the control group missed the follow-up visit at 2 weeks but showed up at 4 weeks, making the numbers of evaluated patients at 2 weeks one less. Table 2 presents baseline characteristics and clinical data for the groups. There were no significant differences between groups at baseline.

**Table 2:** Baseline characteristics of the intervention groups and control group

Characteristic	Intervention groups		Control group
	Active 2^a^ (*n* = 21)	Active 1^b^ (*n* = 25)	Placebo^c^ (*n* = 24)
Age (years), mean (SD)	56.6 (10.3)	64.9 (11.5)	63.1 (11.1)
Sex, *n* (%)			
Female	15 (71.4)	21 (84.0)	14 (58.3)
Male	6 (28.6)	4 (16.0)	10 (41.7)
Nicotine, *n* (%)			
Yes	2 (9.5)	1 (4.0)	6 (25.0)
No	19 (90.5)	24 (96.0)	18 (75.0)
Medical history, *n* (%)			
Hypertension	6 (28.6)	6 (24.0)	8 (33.3)
Hypothyroidism	5 (23.8)	3 (12.0)	6 (25.0)
Asthma	1 (4.4)	2 (8.0)	4 (16.7)
Osteoarthritis	3 (14.3)	5 (20.0)	3 (12.5)
Lichen Ruber planus	1 (4.8)	0 (0)	0 (0)
Genital lichenoid disease	2 (9.5)	2 (8.0)	2 (8.3)
Xerostomia	7 (33.3)	14 (56.0)	9 (37.5)
Depression	0 (0)	2 (8.0)	1 (4.2)
Gastrointestinal disorders	3 (14.3)	4 (16.0)	4 (16.7)
Clinical features of OLP, *n* (%)			
Reticular (only)	0 (0)	0 (0)	0 (0)
Erythematous	17 (81.0)	17 (68.0)	17 (70.8)
Ulcerative	4 (19.0)	8 (32.0)	7 (29.2)
Candida	2 (9.5)	10 (40.0)	7 (29.2)
Localization of OLP, *n* (%)			
Buccal mucosa	21 (100.0)	24 (96.0)	23 (95.8)
Gingiva	19 (90.5)	24 (96.0)	22 (91.7)
Buccal mucosa and gingiva^d^	19 (90.5)	23 (92.0)	21(87.5)
Tongue	10 (47.6)	18 (72.0)	11 (45.8)
Lips	11 (52.4)	14 (56.0)	13 (54.2)
Palate	13 (61.9)	7 (28.0)	9 (37.5)
Mouth floor	10 (47.6)	8 (32.0)	5 (20.8)

^a^Active 2 group: Twice-daily rinse with clobetasol propionate 0.025% oral gel after oral hygiene procedures

^b^Active 1 group: Once-daily rinse with clobetasol propionate 0.025% oral gel and once-daily rinse with placebo oral gel after oral hygiene procedures

^c^Placebo group: Twice-daily rinse with placebo oral gel after oral hygiene procedures

^d^Patients with concurrent lesions in the buccal mucosa and the gingiva

### Primary outcome measure

Table 3 presents the ODSS results. The overall mean ODSS at baseline was 26.4 (± 9.6) with no significant difference between the three groups. Significant reductions in the ODSS occurred in both intervention groups compared to the control group (*p* < 0.05). The degree of reduction in the two intervention groups was similar with no significant differences. At 4 weeks, the ODSS was not significantly better than at 2 weeks. The reduction in the ODSS was significant in all groups after 4 weeks compared to baseline (*p* < 0.05). Differences in the ODSS between groups were primarily due to reductions in clinical signs, rather than reductions in pain scores.

**Table 3:** Primary and secondary outcome measures for the intervention groups and the control group at baseline, and at 2 and 4 weeks

	Intervention groups		Control group
	Active 2^a^ (*n* = 21)	Active 1^b^ (*n* = 25)	Placebo^c^ (*n* = 24^+^)
ODSS, mean (SD)			
Baseline	25.3 (10.2)	26.1 (9.0)	27.6 (9.8)
2 weeks	14.1 (5.7)**	15.0 (6.7)**	23.6 (11.6)
4 weeks	13.5 (6.9)**	15.1 (7.6)*	21.9 (10.0)
Pain score, mean (SD)			
Baseline	2.3 (2.4)	2.3 (2.2)	3.3 (2.4)
2 weeks	1.5 (2.3)	1.1 (1.4)	2.4 (2.4)
4 weeks	0.9 (2.1)	0.7 (1.0)	1.9 (2.2)
Burning sensation score, mean (SD)			
Baseline	4.0 (2.8)	3.9 (2.5)	4.6 (2.5)
2 weeks	1.7 (2.2)	1.6 (1.6)	2.6 (2.5)
4 weeks	1.3 (2.0)	1.0 (1.2)	2.5 (2.4)
OHIP-14, mean (SD)			
Baseline	14.7 (10.1)	14.5 (9.7)	14.5 (9.1)
2 weeks	6.2 (9.0)	5.8 (5.1)	7.9 (6.1)
4 weeks	6.0 (9.2)	4.1 (4.1)	8.3 (7.8)
GRC, % improved			
Baseline	NA	NA	NA
2 weeks	81.0	72.0	47.8
4 weeks	81.0 *	84.0 *	41.7

*OHIP-14* Oral Health Impact Profile − 14 item questionnaire, *GRC* Global Rating of Change, *SD* standard deviation, *NA* not applicable, *ODSS* Oral Disease Severity Score

^a^Active 2 group: Twice-daily rinse with clobetasol propionate 0.025% oral gel after oral hygiene

^b^Active 1 group: Once-daily rinse with clobetasol propionate 0.025% oral gel and once-daily rinse with placebo oral gel after oral hygiene

^c^Placebo group: Twice-daily rinse with placebo oral gel after oral hygiene

^+^At 2 weeks *n* = 23

*p < 0.05, **p < 0.01, difference compared to the control group

### Secondary outcome measures

At baseline, mean overall VAS scores for pain and burning sensation were 2.6 (± 2.3) and 4.2 (± 2.6) with no significant differences between any of the groups (Table 3). The VAS scores did not differ significantly between the intervention and control groups, at neither 2 nor 4 weeks. Compared to baseline, however, VAS scores for pain and burning sensation decreased significantly in all groups (*p* < 0.05).

The mean overall score for OHIP-14 at baseline was 14.6 (± 9.5). At baseline and at 2 and 4 weeks, there were no significant between-group differences in the OHIP-14 score. However, compared with baseline, the intervention and control groups experienced a significant reduction in the OHIP-14 score at 2 weeks (*p* < 0.001). The Active 1 group also experienced a significant reduction in the score between weeks 2 and 4 (*p* < 0.01) while the other groups did not.

At 4 weeks, perceived improvement according to the GRC was significant in the two intervention groups compared to the control group (*p* < 0.05). There were no differences between the two intervention groups neither at 2 weeks nor at 4 weeks (Table 3).

### Adverse effects

The adverse effects reported by the patients were mild and occurred in all three groups with no significant differences. The most reported adverse effects were gastrointestinal symptoms (19%), dysgeusia (10%), dysesthesia (10%), and burning sensation (6%). Gastrointestinal symptoms were primarily gastric, with fewer symptoms of intestinal origin. No adverse events that could be associated with glucocorticoid-induced adrenal insufficiency such as fatigue, muscle weakness, dizziness, nausea, or low mood were reported in either active group.

**Fig. 2:**
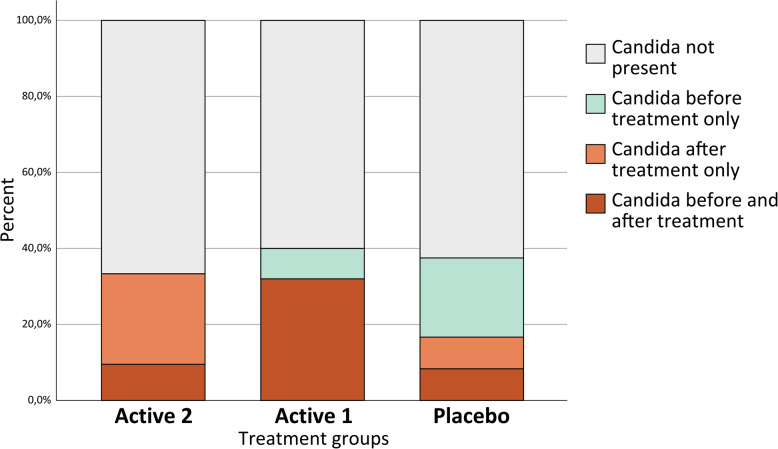
Presence of Candida in mucosal smears, before and after treatment. Active 2: Twice-daily rinse with clobetasol propionate 0.025% oral gel; Active 1: Once-daily rinse with clobetasol propionate 0.025% oral gel and once-daily rinse with placebo oral gel; Placebo: Twice-daily rinse with placebo oral gel

No obvious clinical signs of oral candidiasis were observed during the study, however candida hyfae was detected in the mucosal smears from the OLP lesions. At baseline, oral candidiasis in OLP lesions was present in 19 (27%) patients, distributed evenly across all three groups. After 4 weeks, the total number of cases remained unchanged. However, the distribution shifted during the study, with some cases resolving and new cases emerging. In the control group, two new cases occurred in patients with poor adherence to antifungal prophylaxis. Among patients who adhered to prophylaxis and were negative at baseline, five new cases occurred, all in the Active 2 group (*p* < 0.05; Fig. 2).

## Discussion

In this randomized, placebo-controlled trial of symptomatic OLP, once-daily treatment with clobetasol 0.025% was as effective as twice-daily treatment. Both regimens resulted in significant improvement in the ODSS and the GRC scores compared with placebo. These findings support a reduced-frequency, reduced-concentration clobetasol regimen. It may serve as a suitable treatment approach for newly diagnosed patients with symptomatic OLP.

Although topical corticosteroids are considered first-line therapy, evidence supporting optimal dosing strategies has been limited, particularly in the short term. Three previous randomized placebo-controlled trials assessing topical corticosteroids for OLP have demonstrated clinical improvement with fluocinolone, triamcinolone, and clobetasol formulations [13–15]. However, treatment frequency, formulation and mode of evaluation have varied across these studies. There are several disease scoring systems for OLP [27]. One of the most used scoring systems is the Thongprasom index [17]. Though widely used and practical, this index offers a less detailed picture of the clinical situation and does not include experienced symptoms. Since the ODSS has shown clinical sensitivity and high reproducibility [28], and has been validated for OLP [29], we found it suitable for assessing treatment outcomes. The RCT study by Arduino et al. [15] found that clobetasol 0.05% twice daily reduced median clinical scores according to the Thongprasom index from 4.0 at baseline to 2.0 at week 4, a 50% decrease, with no further improvement between weeks 4 and 8. Although a different method for clinical scoring (ODSS) was used in the present study, a comparable reduction was observed. Further, this reduction was achieved at 2 weeks using clobetasol 0.025% once daily.

Several non-placebo controlled RCTs have investigated the effect of clobetasol on OLP and most of them have used a concentration of 0.05% [20]. However, a study by Carbone et al. found no statistically significant differences in treatment outcomes between clobetasol 0.05% and 0.025% in OLP [35]. Studies on inflammatory dermatologic conditions have shown that clobetasol 0.025% has an efficacy comparable to 0.05% while being associated with fewer adverse events [36, 37]. Several different preparations are available for local corticosteroid treatment of OLP, including ointments, gels for rinsing, mucoadhesive patches, and local injections. In Sweden, clobetasol 0.025% oral gel for rinsing has been used since 2007, with empirically documented positive outcomes [38].

Although both active treatment groups in this study demonstrated improvements in clinical signs compared with placebo from week 2 onward, patient-reported outcomes were more varied. The patients’ perceived overall change following treatment, as measured by the GRC, favoured active treatment over placebo but was not evident until week 4. This delayed symptom response suggests that the clinical signs may improve earlier than symptom perception, which is relevant when interpreting short-term treatment effects. No differences were observed between active groups and placebo for pain, burning sensation, or OHIP-14 scores, and all groups improved to a similar extent. These values were relatively low at baseline, potentially reducing the sensitivity to detect clinically meaningful changes which could have contributed to an underestimation of clobetasol’s effect on symptom relief. This pattern may reflect the challenge of detecting domain-specific changes in OLP studies. Many commonly used patient-reported outcome measures, such as the OHIP, have limited validated interpretability and do not capture multidimensional outcomes [39, 40]. Measures of perceived overall change following treatment, such as the GRC, may therefore reflect overall clinical benefit more effectively than isolated symptom scales [33].

The control group showed substantial improvement in patient-reported outcome measures. Earlier studies on local corticosteroid treatment, including clobetasol, report similar effects [13–15, 41]. A possible explanation could be that contextual factors, such as patient-clinician interaction and patient expectations, may have influenced the results. Further, the act of rinsing six times daily, regardless of the pharmacological properties of the substances used, could have influenced the outcomes, particularly in patients experiencing oral dryness.

A significant improvement in objective clinical signs (ODSS) was observed in the control group. Similar findings have been reported in earlier OLP trials [13–15] and likely reflect the fluctuating natural course of OLP [42] and regression to the mean [43]. Although the ODSS includes structured scoring criteria, minor observer effects cannot be entirely excluded, even in double-blind studies when outcome assessment involves clinical judgement [44]. In addition, all participants received antifungal prophylaxis, which represents a potential confounder when interpreting lesion improvement in the control group.

Despite antifungal prophylaxis, cytologically confirmed oral candidiasis in OLP lesions was observed more frequently among patients receiving clobetasol, particularly in the twice-daily group, where five new cases occurred. Although most cases were mild, this pattern suggests a dose-related increase in susceptibility to fungal overgrowth during topical corticosteroid treatment. It also indicates that nystatin, at the administered dose, may be insufficient. This aligns with observations from Marable et al. [45], who reported that candidiasis occurred relatively frequently in patients receiving corticosteroid therapy and that prophylaxis may reduce but not eliminate the risk of fungal overgrowth.

The present study has some limitations. While inclusion was restricted to patients meeting the modified WHO criteria for OLP, oral lichenoid lesions, including drug-related and contact-related variants, may be clinically and histopathologically indistinguishable from OLP. In the absence of additional investigations such as patch testing, it cannot be excluded that some patients may have had concurrent or misclassified lichenoid lesions. The relatively short treatment period reflects ethical constraints associated with placebo use in symptomatic OLP but may have limited the ability to detect long-term differences between groups. Previous placebo-controlled trials regarding corticosteroid treatment of OLP with symptoms have typically evaluated treatment durations of 4–8 weeks [13–15] and disease activity is known to fluctuate over time [42]. As a result, short-term studies primarily capture initial anti-inflammatory effects rather than sustained remission or recurrence [12]. Another limitation was that all patients received antifungal prophylaxis. This routine may have reduced the risk of corticosteroid-related candidiasis but may also have influenced mucosal healing by modifying the local microbial environment [45]. On the other hand, it cannot be fully excluded that fungal hyphae within OLP lesions may have affected patient-reported symptoms, even in the absence of clinically evident oral candidiasis. Dysesthetic or neuropathic-type oral symptoms have also been described in patients with OLP and may not always correlate with the degree of mucosal inflammation [46]. Such characteristics were not systematically assessed in the present study. However, regardless of any sensory alteration, a clear improvement in patient-reported outcomes was observed compared to baseline in all groups. Lastly, the study sample was too small to detect significant differences in the secondary outcome measures (pain, burning sensation, and the OHIP-14) between the two active regimens and placebo.

## Conclusion

The findings indicate that 0.025% clobetasol propionate oral gel is superior to placebo in reducing disease severity, as measured by the Oral Disease Severity Score (ODSS), and in improving self‑reported symptoms, as evaluated with the Global Rating of Change (GRC) scale, in patients with symptomatic OLP. The results further suggest that once‑daily administration is as effective as twice‑daily dosing.

## Data Availability

The datasets used and/or analysed during the current study are available from the corresponding author upon reasonable request.

## Abbreviations

GRC: Global Rating of Change
ODSS: Oral Disease Severity Score
OHIP-14: Oral Health Impact Profile-14
OLP: Oral Lichen Planus
RCT: Randomized Controlled Trial
VAS: Visual Analog Scale
